# GWAS of Active Music Engagement Frequency in the Canadian Longitudinal Study on Aging

**DOI:** 10.21203/rs.3.rs-8108581/v1

**Published:** 2026-01-23

**Authors:** Tara Henechowicz, Brooke Wolford, Rachana Nitin, Yasmina Mekki, Alyssa Scartozzi, Peyton Coleman, Earvin Tio, Grace Schlicht, Srishti Nayak, Michael Thaut, Daniel Felsky, Reyna Gordon

**Affiliations:** University of Toronto; Centre for Addiction and Mental Health; Centre for Addiction and Mental Health

**Keywords:** GWAS, musicality, aging, music, music engagement, cognition, mental health, psychiatric disorder risk, motor function, language, partitioned heritability, genetic correlations, Mendelian Randomization, CLSA

## Abstract

Active music engagement (AME), i.e., playing a musical instrument or singing, is moderately heritable and may support resilience to age-related functional decline. To understand AME’s genetic architecture, we conducted a genome-wide association study (GWAS) in the Canadian Longitudinal Study on Aging (n = 23,782 with genetically inferred European ancestry). SNP-based heritability was estimated at 10%, revealing 21 independent loci at suggestive significance (*p* < 5×10^−5^). Post-GWAS analyses showed enrichment in regulatory regions of adult brain cells and genetic correlations with musical rhythm ability, language, and cognition. Secondary genetic correlation analyses (bivariate-GREML) linked AME to enhanced cognition, motor function, social engagement, and resilience to psychological distress, but also increased mood disorder risk. Lastly, bi-directional Mendelian randomization indicated that individuals who have greater genetic propensity for musical rhythm abilities are more likely to have more frequent musical instrument or singing engagement. Overall, these findings suggest that the polygenic architecture of AME is enriched for neurobiological function, specifically promoter of astrocyte function, and shares genetic variation with healthy aging.

## Introduction

Playing a musical instrument or singing is a cognitively stimulating, social, and physically demanding activity^1^. Most music and aging research has focused on music-based interventions (MBIs), which have promising effects on cognition, motor function, and social-emotional well-being in elderly patients suffering from cognitive decline^2^. In parallel, active music engagement frequency, i.e., how much people play a musical instrument or sing in everyday life, may be viewed as a modifiable lifestyle factor that, like exercise frequency, predicts several aspects of healthy aging^3–6^. In older adults, several cross-sectional studies have shown differences between older musicians and non-musicians in cognitive and motor function, e.g., sensorimotor synchronization, non-verbal visual memory, executive function, and auditory attention^7^. Mechanistically, it is hypothesized that music training throughout life contributes to enhanced cognitive resilience despite potential neurodegeneration^8^.

Recent population-level investigations of music engagement in aging adults further demonstrate connections between music in everyday life and health outcomes. For example, a recent longitudinal investigation from the Lothian Birth Cohort showed that musical instrument engagement predicts improved cognitive function in older adults^9^. Similarly, arts activities involving active (e.g., crafting, choir, or dance groups) as opposed to receptive involvement (e.g., going to a concert or going to the museum) are associated with lower risk for chronic health outcomes and cognitive decline^10^ and greater frequency of participating in community arts groups (e.g., choir, dance, photography, theatre, or music groups) are associated with greater life satisfaction and wellbeing^11^.

Playing musical instruments or singing in everyday life varies significantly across the population and is influenced by genetic factors. On average, twin-based studies have shown 42% heritability of musical behaviours^12^. The heritability of active musical instrument engagement varies across the lifespan, with low heritability (or primarily environmental contributions) for children ^13^ and moderate to high heritability in adolescents and adults (e.g., instrument engagement in adults: 78%; singing engagement in adolescence: 43% ^14^; total lifetime hours of practicing a musical instrument in adults: 40–70% ^15,16^.

Most prior genetic investigations of active music engagement have yet to characterize the molecular mechanisms of known genetic influences of active music engagement. Genome-wide association studies (GWAS) complement twin designs and provide important insights into the biology of active music engagement and its shared etiology with a range of health traits. A well-powered GWAS of musical rhythm abilities (specifically, beat synchronization) in 606,825 adults aged 18 to 60 + years old) identified 69 significant loci and showed 13–16% SNP-based heritability. Further, musical rhythm abilities were enriched for adult brain-specific gene regulatory elements and were genetically correlated with several health functions, including biological rhythms (e.g., breathing; chronotype), motor function (e.g., walking pace), and cognitive function (e.g., processing speed)^17^. Given the relevance of music-related traits for the brain, biology, and health, more genome-wide investigations of several dimensions of musical behaviours, like active music engagement, are needed specifically in older adult populations.

Here, we use GWAS approaches to map the neurobiological underpinnings of active music engagement in aging, identify epidemiological associations with aging-related health traits, and build mechanistic explanations for any associations. Our approach was as follows: (1) we conducted a GWAS of active music engagement frequency in the Canadian Longitudinal Study on Aging (CLSA; n = 23,782, ages 45 to 85 with European ancestry); (2) we further examined the neurobiological function of the genetic architecture of active music engagement frequency by estimating SNP-based heritability, using functional annotations of eQTLs for brain tissues to map SNPs genes, and conducting enrichment analyses with neurobiological gene sets; (3) we validated GWAS results by calculating polygenic scores in an independent cohort with similar music engagement phenotypes; (4) we investigated genetic correlations between active music engagement and health traits using two different methods; and (5) we conducted bidirectional two-sample Mendelian randomization (MR) studies to explore causal associations between active music engagement, beat synchronization, and language to understand health implications further. These analyses demonstrate how active music engagement in aging has genetic influences that are functionally intertwined with neurobiological functioning, are genetically correlated with several domains of healthy aging, and may be caused by genetics of musical rhythm abilities. Our results have implications for shifting our understanding of “music and health” to promoting music engagement as an indicator of healthy aging.

## Results

An overview of the study’s methodology, analytical flow, and data resources. GWAS=genome-wide association study; SNP=Single Nucleotide Polymorphism; LDSC=Linkage Disequilibrium Score regression analysis pipelines ^18^; Bivariate-GREML=Bivariate-Genome-based restricted maximum likelihood method for estimating the shared genetic covariance between two traits as implemented in the GCTA software ^19^; eQTL=expressive quantitative trait loci; CLSA=Canadian Longitudinal Study on Aging. AME=Active music engagement. Dotted arrows in the Mendelian Randomization schema represent the tested causal pathways, however the red arrow represents the causal direction with significant results. Figure created using Biorender.com.

### Sample Demographics

**Figure 2:**
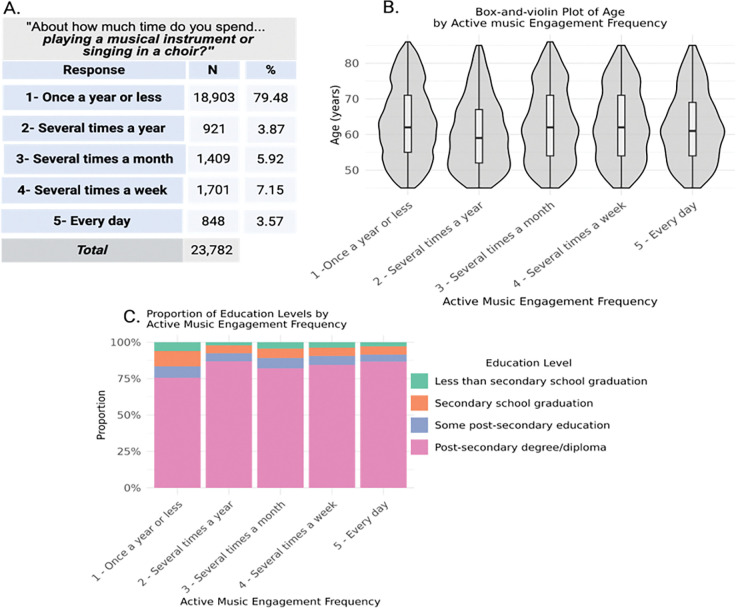
Sample Demographics *Note.* The figure shows the phenotype definition and sample demographics, including (A) the distribution of the phenotype for active music engagement frequency and the phenotype definition, (B) the age distributions for each level of active music engagement frequency expressed as box-violin plots, and (C) the distribution of education for each level of active music engagement frequency. The figure was created using Biorender.com.

The median age of participants was 62.0 years (interquartile range [IQR]=55,71), and the mean age was 63 ±10.17 years, and 11,949 (50.24%) of the sample were female. The sample was highly educated, with n=18,388 (77.32%) having at least a post-secondary degree or diploma. Active music engagement frequency had significant negative correlations with age (r_τ_ = −0.03, z=−5.4, *p* < .001) and positive correlations with education levels (r_τ_ = 0.08, z=13.58, *p* < .001), showing that individuals who played a musical instrument or sing in a choir more frequently tend to be younger and more educated. See Figure 2. for the phenotype distribution and the sample demographics.

### GWAS Results

**Figure 3:**
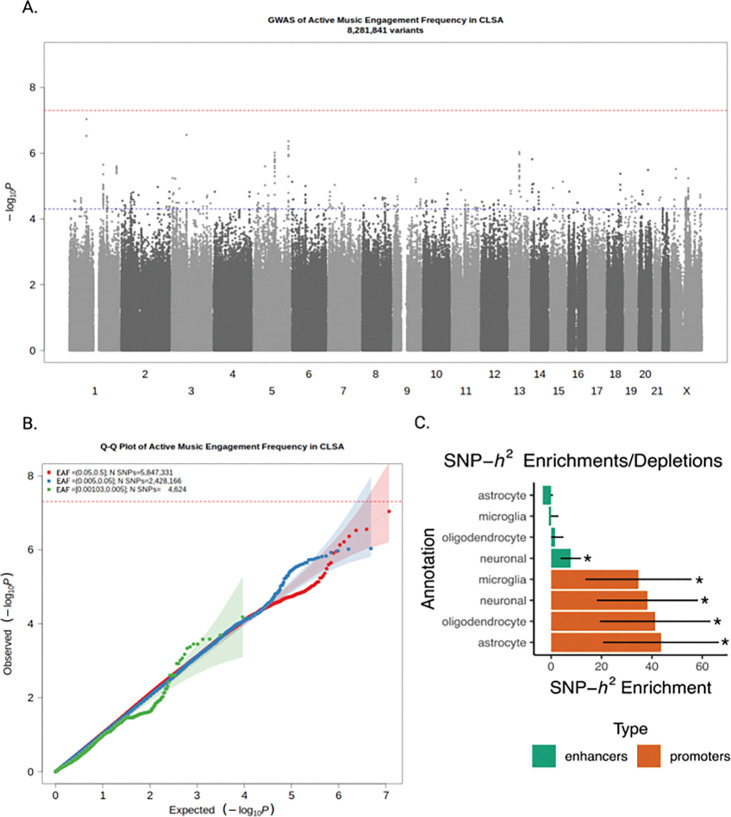
Results of the GWAS of Active Music Engagement Frequency *Note.* A) Manhattan plot showing SNP associations (−log10(*p*-value)) with active music engagement frequency, ordered by chromosome. The red dashed line indicates the threshold for conventional genome-wide significance (*p*=5×10^−8^), and the blue dashed line indicates the threshold for suggestive significance (*p=*5×10^−5^). B) The Q-Q Plot represents a comparison of the *p*-values of the GWAS by the *p*-values expected for null distribution (depicted by the dotted grey line). The p-values for the GWAS are binned by effect allele frequency (EAF). C) The bar plot represents the SNP-h2 Enrichment estimates for each category, with error bars as standard errors. (*) Denotes significant enrichment in that category of annotations (*q*FDR<0.05).

We conducted a GWAS of active music engagement frequency in n=23,782 individuals of genetically inferred European ancestry in CLSA with n=8,321,411 common SNPs. Active music engagement frequency was measured as a single self-reported item in the CLSA (see Figure 2A. and **Supplementary Methods 1.** for phenotype definition). Genomic inflation was mild and likely due to polygenicity rather than issues in population stratification (χ2=1.05, λ_GC_=1.04, the LD score intercept was 1.03(SE=0.0064), and the ratio was 0.55(SE=0.14), see Figure 3b. for the Q-Q plot).

Although our GWAS did not reveal any significant SNPs at the genome-wide threshold of *p*<5×10^−8^, our results showed a polygenic signal with strong linkage disequilibrium and potential suggestive loci (See Figure 3a. for the Manhattan plot). For follow-up functional enrichment analysis, we carried forward all SNPs significant at a suggestive threshold of *p<*5×10^−5^, which yielded 28 independent lead SNPs within 21 loci after LD clumping.

Functional analyses using ANNOVAR revealed that the functional consequences of all independent significant SNPs (and those within linkage disequilibrium) were primarily in non-coding regions, with 79.8% having intergenic function. Using eQTL reference data from GTEx (v8), Schwartzentruber et al.’s (2018) annotations for sensory neuron function, xQTL dorsolateral prefrontal cortex tissues, PsychENCODE, BRAINEAC, and CommonMind Consortium, we identified 23 genes with expression regulated by suggestive loci, six of which were expressed in brain tissues (See Table 1. and methods xx). Notably, a genomic locus on chromosome 1 had three eQTLs affecting gene expression in the cerebellum, including two independent significant SNPs, which were the two top hits of the GWAS (See **Supplementary Figure 1** for locus zoom plot). The A allele of lead SNP rs7554669 (frequency=0.18) was associated with less frequent music engagement (𝛽=−0.07, *p*=9.20×10^−8^) and is linked to lower expression of a region of long non-coding RNA, *RP11–131L23.1,* in the cerebellum (𝛽=−0.40, *p*=6.12×10^−5^, *q*FDR=0.013, GTExV8). The functions of *RP11–131L23.1* are largely unknown, although in general, long non-coding RNA could have several downstream effects on gene expression ^20^. Five additional genomic loci had eQTL mappings affecting gene expression in brain tissues (See Table 1). An additional locus on chromosome 15 has six eQTL-mapped genes (see Table 1. and **Supplementary Figure 17**) including the lead SNP, rs4572341^A^ (frequency=0.09, 𝛽=0.08, *p=*7.3×10^−6^) that was an eQTL affecting affects expression of *RP11–561C5.4* in brain tissue (psychENCODE), adipose (GTEx v8), and lung tissues (GTEx v8), *CSPG4P12* in skeletal muscle tissue (GTEx v8), and *RP11–815J21.3* and *RP11–158M2.5* in testis tissue (GTEx v8). See **Supplementary Tables 3–8** for FUMA results and **Supplementary Figures 1–21** for locus zoom plots.

### Heritability

We investigated the SNP-based heritability of active music engagement to understand the relative contribution of common genetic variation to variability in active music engagement within an aging population. The GCTA-GREML ^21^ estimated heritability of active music engagement frequency was 10% (*h*^2^_SNP_ =0.10, *p*=1.16×10^−9^, 95% CI [0.06, 0.14], power =1, n=19,522), in line with complex polygenic traits and aligning with previous estimates, e.g., 12% for music engagement in Vanderbilt’s Online Musicality study (age=44.90 ±16.24 years) ^22^. To investigate the neurobiological function of the genetic variation of active music engagement frequency in aging, we performed LDSC partitioned heritability analyses using cell-type specific annotations of promoter and enhancer regions of neurons, microglia, astrocytes, and oligodendrocytes ^23^. Partitioned heritability analyses showed significant enrichment in neuronal promoter (Enrichment(SE)=38.17(20.05), *p=*0.003 *q*FDR=0.007) and enhancer regions (Enrichment(SE)=7.76(4.11), *p=*0.029, *q*FDR=0.047), as well as in promoter regions of astrocytes (Enrichment(SE)=43.57(22.96), *p=*0.002, *q*FDR=0.007), oligodendrocytes (Enrichment(SE)=41.29(21.94), *p=*0.002, *q*FDR=0.007), and microglia (Enrichment(SE)=34.65(21.05), *p=*0.024, *q*FDR=0.047). See Figure 2C. and **Supplementary Table 9**. These results suggest that the common genetic variation associated with active music engagement frequency in aging is also implicated in regulatory functions of brain cell types and important brain structures. These results are consistent with previous findings of the GWASs of musical rhythm, dyslexia, the multivariate GWAS of rhythm impairment and dyslexia ^24^, where there was significant enrichment for multiple brain cell types including with the greatest enrichment for promoter regions of neuronal cells and oligodendrocytes. Our results also showed a similar signature to the partitioned heritability of general cognitive function ^23^, while also contrasting that of SNP-based heritability of active music engagement contrasts that of the GWAS of Alzheimer’s disease, which showed significant enrichment for microglial enhancers but not for any other regulatory regions of cell types ^23^.

### Polygenic Score Replication Studies

We investigated whether polygenic scores (PGS) derived from the GWAS of active music engagement frequency (PGS_music_) calculated using PRS-CS ^25^ predicted active music engagement in two waves of data within an external aging cohort, Wisconsin Longitudinal Study (WLS). In the “2003–2005” wave (mean age=64.23±2.51, 51% female, N_cases_=543, N_controls_=4301), a higher PGS_music_ was associated with a greater likelihood of practicing a musical instrument (OR = 1.13 per s.d. increase in PGS_music_, 95%CI [1.02,1.24], *p*=0.01, Nagelkerke-*R*^*2*^=0.03). In the “2011” wave (mean age=70.88±2.55 years, 53% female, N_cases_=450, N_controls_=3556), a higher PGS_music_ was associated with a greater likelihood of practicing a musical instrument (OR = 1.25 per s.d. increase in PGS_music_, 95%CI [1.13,1.39], *p*<0.001, Nagelkerke-*R*^*2*^=0.05). These results demonstrate that the genetic propensity for active music engagement frequency in aging can significantly predict a related active music engagement phenotype in new participants, with small yet non-trivial effect sizes.

**Figure 4:**
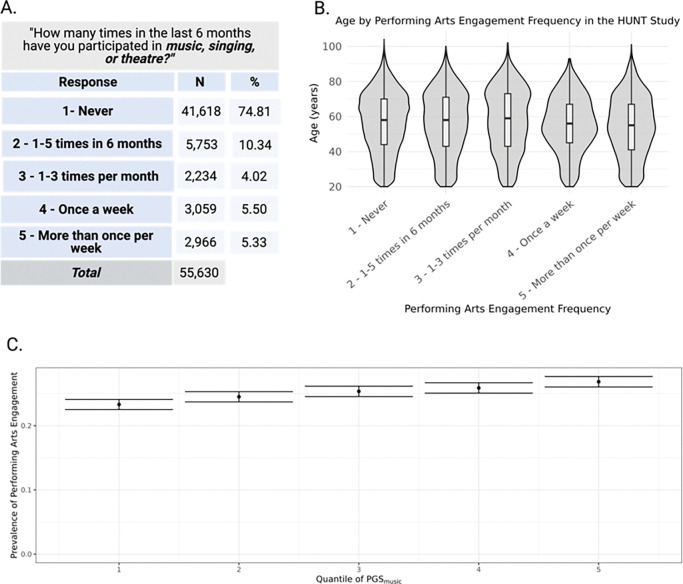
Results of the PGS replication study in the HUNT study *Note.* The figure shows (A) the distribution of performing arts engagement in the Trøndelag Health Study demographics, (B) the age distributions for each level of performing arts engagement expressed as box-violin plots, and (C) the distribution of education for each level of active music engagement frequency. The figure was created using Biorender.com.

Additionally, we sought to replicate findings within a large general population sample. Therefore, we examined associations whether polygenic scores PGS_music_ predicted performing arts engagement (i.e., music, singing, or theatre engagement) in the n=56,216 from the Trøndelag Health Study (HUNT) (mean age = 56.27±17.61, 53% female, see Figure 4A for the phenotype distribution and Figure 4B for the age distributions for each level of performing arts engagement). A higher PGS_music_ was associated with more frequent participation in music, singing, or theatre activities within the past 6 months (beta= 0.037 per s.d. increase in PGS_music_, 95%CI [0.027,0.046], *p*=4.65E-14, Nagelkerke-*R*^*2*^=0.002). Further, we have visualized the prevalence of those who engage in performing arts (i.e., those who engage at least 1−-5 times in six months or greater versus never) in different quintiles of PGS_music_, showing that the prevalnce of engagement increased with higher PGS_music._ (See Figure 4C). In summary, these results of our polygenic score replication studies suggest that the genetic propensity for active music engagement frequency in aging predicts active music engagement-related phenotypes in an external aging cohort and a general population study.

### Genetic Correlation Analyses

**Figure 5:**
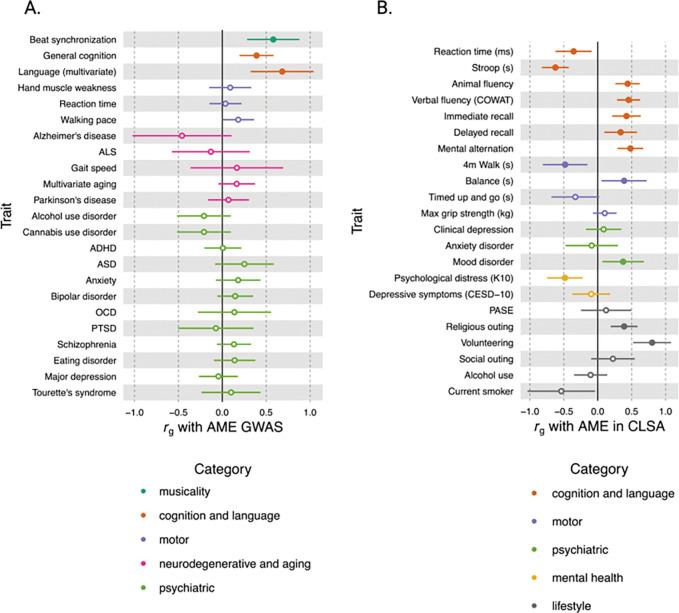
Genetic Correlations between Active Music Engagement Frequency in Aging and Health Traits *Note.* (A) GWAS-based genetic correlations between active music engagement frequency and 23 aging-related health traits (estimated using LDSC) with 95%CI. The genetic correlation with multiple sclerosis is not pictured due to large standard error. (B) Bivariate-GREML genetic correlations between active music engagement frequency and aging-related health traits. For both figures, filled-in circles are significant (*q*FDR *<*0.05), and open circles are not significant. COWAT=Controlled Oral Word Association Task; K10=Kessler Psychological Distress Scale; CESD-10=Center of Epidemiologic Studies Depression Scale, 10-item version; PASE=Physical Activity Scale for the Elderly.

We investigated genetic correlations between music engagement frequency in aging and 24 health-relevant phenotypes with existing external GWASs using LDSC and phenotypes within the CLSA cohort using bivariate GREML. LDSC genetic correlations were conducted with a range of GWASs aging processes, neurodegeneration, psychiatric diagnoses, cognition, language, motor function, and musical rhythm (i.e., beat synchronization) (see **Supplementary Table 1.** for the source GWASs and complete results). As expected, the results revealed evidence for shared genetic architecture of active music engagement and musical rhythm abilities, i.e., there were significant correlations between the GWAS of active music engagement frequency and beat synchronization (*rg*=0.58, 95%CI [0.28, 0.88], *p=*1×10^−4^, *q*FDR=0.0012). Additionally, we observed significant positive genetic correlations with general cognitive function (*rg*=0.39, 95%CI [0.20, 0.58], *p=*7.7×10^−5^, *q*FDR=0.0012) and multivariate GWAS of language abilities (*rg*=0.68, 95% CI[0.32,1.04], *p*=2×10^−4^, *q*FDR=0.0016) (see results in Figure 5A), further supporting evidence for shared etiology of language abilities and musicality^24,26^.

We conducted complementary analyses of bivariate-GREML genetic correlations with active music engagement frequency and similar health traits available in CLSA to understand the shared genetic influences specific to aging since prior GWAS-based analyses were not restricted to aging populations (see Figure 5B. and complete results in **Supplementary Table 2.)**. First, the bivariate-GREML analyses showed significant genetic correlations between active music engagement frequency and higher cognitive function. We observed genetic correlations between higher music engagement frequency and faster processing speed (reaction time in milliseconds, *r*_*g*_*=−*0.36, 95%CI [−0.62, −0.09], *p=*0.009, *q*FDR=0.02), enhanced executive functioning (Stroop interference task performance, *r*_*g*_*=−*0.63, 95%CI [−0.82, −0.43], *p*=5.68×10^−6^, *q*FDR=1.25×10^−8^), preserved verbal fluency (Animal Fluency task, *r*_*g*_*=*0.44, 95%CI [0.26, 0.62], *p*=1.71×10^−6^, *q*FDR=7.54×10^−6^; Controlled Oral Word Association Task, *r*_*g*_*=*0.45, 95%CI [0.29, 0.62], *p*=1.44×10^−7^, *q*FDR=1.06×10^−6^), preserved memory function for immediate recall (Rey Auditory Verbal Learning Test-immediate recall, *r*_*g*_*=*0.42, 95%CI [0.21, 0.63], *p=*8.02×10^−5^, *q*FDR=0.0003), delayed recall (Rey Auditory Verbal Learning Test-delayed recall, *r*_*g*_=0.34, 95%CI [0.10, 0.58], *p=*0.006, *q*FDR=0.01), and greater mental flexibility and processing speed (Mental Alternation Test, *r*_*g*_*=*0.48, 95%CI [0.29, 0.67], *p*=4.94×10^−7^, *q*FDR=2.72×10^−6^). Together, both methods of genetic correlations showed robust evidence for significant shared genetic architecture between music engagement frequency and beat synchronization, cognition, and language traits.

Additionally, bivariate-GREML resuts revealed significant genetic correlations between higher active music engagement frequency and better motor function, i.e., better balance (best balance time in seconds, *r*_*g*_*=*0.39, 95%CI [0.06, 0.72], *p=*0.02, *q*FDR=0.04) and faster gait speed (four-metre walk test in seconds, *r*_*g*_*=−*0.48, 95%CI [−0.81, −0.15], *p=*0.004, *q*FDR=0.01), and greater social participation, i.e., going out to religious activities ( *r*_*g*_*=*0.39, 95%CI [0.19, 0.59], *p=*0.0001, *q*FDR=0.0004) and volunteering (*r*_*g*_*=*0.80, 95%CI [0.52, 1.08], *p=*1.90×10^−8^, 2.09×10^−7^). We also observed significant genetic correlations between greater active music engagement frequency and lower psychological distress (Kessler Psychological Distress Scale, *r*_*g*_*=−*0.48, 95%CI [−0.75, −0.22], *p*=0.0003, *q*FDR=0.0008), indicating a potential protective effect of active music engagement frequency on resilience to mental health symptoms.

Despite these consistent correlations with better physical and cognitive health, the bivariate-GREML analyses revealed that greater active music engagement frequency was genetically correlated with greater risk for mood disorders (*r*_*g*_*=*0.37, 95%CI [0.07, 0.68], *p=*0.02, *q*FDR=0.03). It is also notable that LDSC-based genetic correlations did not show any significant associations between active music engagement frequency and any psychiatric diagnosis (derived from the Psychiatric Genomics Consortium meta-GWASs). Our results could reflect more complected genetic by environment interactions where those who are likely to engage in music, have heightened genetic risk for psychiatric problems^17,27,28^, yet engaging with music could also reduce psychological distress^29^.

### Bidirectional Two-Sample Mendelian Randomization Studies

Taking forward significant GWAS-based genetic correlations, we conducted bidirectional two-sample Mendelian randomization (MR) studies to explore the causal associations between active music engagement frequency to language ability and active music engagement frequency to beat synchronization. We did not conduct analyses with general cognitive function, given our ethical and methodological concerns of inferring causality ethical with broader measures of cognition (see Box 1). The results for all two-sample MR analyses are in Table 2, sensitivity leave-one-out results in **Supplementary Table 10**).

**Figure 6:**
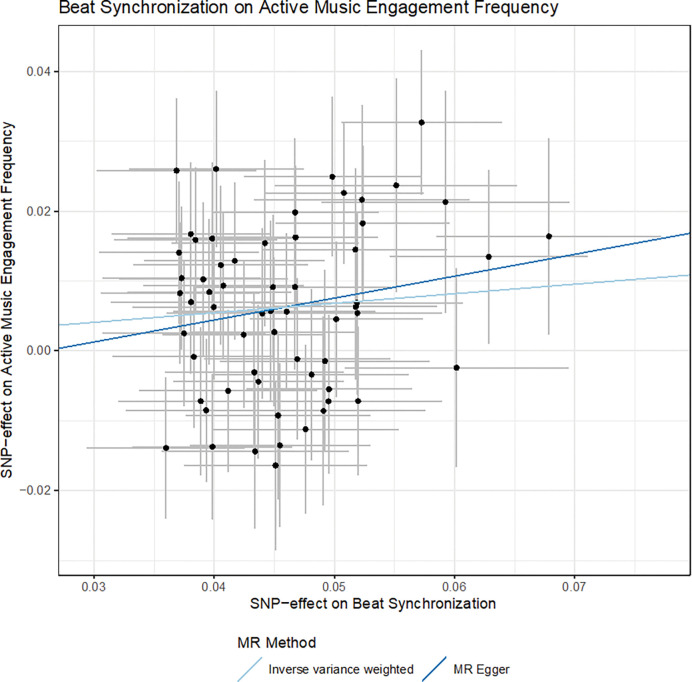
Scatter Plot of the SNP-effects of Musical Beat Synchronization (Exposure) on Active Music Engagement Frequency (Outcome) *Note.* The scatterplot shows the SNP-effects on active music engagement with 95%CIs by the SNP-effect on beat synchronization with 95%CIs. Regression lines are plotted for the meta-analyses using the inverse variance weighted and MR-Egger methods. Overall, results show a positive causal effect of beat synchronization on active music engagement frequency.

The results indicated that the genetic influences of beat synchronization may cause higher music engagement frequency in aging using the inverse-variance weighted method (*b=*0.14, 95%CI [0.07,0.20], *p=*4.2 ×10^−5^) (See Figure 6 for scatter plot and **Supplementary Figures 22–23** for forest plots of leave-one-out analyses). Our results are consistent with previous work showing that PGS for beat synchronization predicts music engagement in external cohorts ^22,30^. Notably, the top independent significant SNP for the beat synchronization GWAS, rs848293 mapped to *VRK2,* had a significant causal effect on active music engagement frequency (*b*=0.57, 95%CI [0.22, 0.93], *p*=0.002). In the beat synchronization GWAS, the effect of rs848293 was *b*=−0.06, SE=0.01, *p*=9.2×10^−18^, EAF=0.58, and in the active music engagement frequency GWAS, the effect of rs848293 was *b*=−0.03, SE=0.01, *p*=0.002, EAF=0.58. While these analyses were not biased by pleiotropy (MR-egger intercept *p*=0.44), we do note that the SNP-exposure correlation was not greater than the SNP-outcome correlation, (SNP-exposure-*r*^*2*^=0.0042, SNP-outcome-*r*^*2*^=0.0041, Steiger test *p*=0.87). While this necessitates caution when interpreting directionality, the Steiger test is not reliable when observed correlations are small and similar ^31^. Nevertheless, our results further our understanding of the connection between these musical behaviours, showing a potential beneficial relationship between increased beat synchronization and more active music engagement. For language ability, results did not reveal any evidence for a causal relationship between active music engagement frequency and language ability using any of the two-sample MR regression methods.

## Discussion

Our GWAS revealed insights into the polygenic architecture of music engagement frequency, showing that it is a neurobiological trait deeply connected to several facets of healthy aging. The top suggestive loci were eQTLs affecting gene expression in the cerebellum, an essential structure for motor timing and musical rhythm ^32–35^. Genetic correlation results suggest that active music engagement shares biological underpinnings with healthy aging, i.e., maintaining cognitive and language function, mental health resilience, motor function, and increased social engagement, despite also showing associations with increased risk for mood disorders. Lastly, beat synchronization may *cause* higher amounts of music engagement, providing the groundwork for understanding the direction of molecular pathways involved in musical behaviours.

Our results provide novel insights, suggesting that the function of the common genetic variation associated with active music engagement frequency is implicated in cerebellar gene expression. Six of the 21 suggestive loci had eQTL-mapped genes that affect gene expression in brain tissues. The top hit of the GWAS, rs7554669, was implicated in affecting gene expression in the cerebellum ^36^. Our findings support those from the GWAS of musical beat synchronization, which was enriched for genes expressed in the cerebellum, dorsolateral prefrontal cortex, inferior temporal lobe, and basal ganglia ^17^. The cerebellum is essential for broader cognitive, motor, and timing functions ^33^ and is also a central node in the musical rhythm network ^35^. Prior neuroimaging studies have shown that adult and older adult musicians, compared to non-musicians, typically exhibit a greater grey matter volume in this cerebellum ^7^. However, this finding did not pass multiple testing corrections in a recent meta-analysis ^37^. Collectively, our results offer supporting genetic evidence for the link between the cerebellum and music engagement, complementing prior neuroimaging studies.

Our partitioned heritability analyses showed that the common genetic variation associated with active music engagement frequency was enriched for promoter and enhancer regions of neuronal cell types and promoter regions of other brain cell types, including oligodendrocytes, astrocytes, and microglia. These analyses suggest that genetic variation at promoter regions influencing cellular processes across the brain gives rise to active music engagement later in life. Likewise, these patterns of enrichment were similar for GWASs of cognitive traits and the common factor of rhythm and language ^24^. Follow-up work should further assess the specific contributions of these cell types to active music engagement and investigate additional regulatory mechanisms.

Genetic correlation analyses and our follow-up bidirectional two-sample MR study shed light on the connections between active music engagement, beat synchronization, and language abilities through a genetic lens. First, evidence from three separate studies has already established genetic associations between beat synchronization and music engagement ^17,22,30^. Our result illustrates the first statistical evidence that these associations are more likely to flow in the direction where beat synchronization *causes* higher active music engagement frequency in aging rather than the reverse. Thus, the genetics of beat synchronization affect active music engagement frequency through *vertical pleiotropy* rather than *horizontal pleiotropy* ^41,42^. Additionally, experimental studies have shown that musicians, compared to non-musicians, have enhanced neuro-facilities for beat synchronization (e.g., auditory-motor connectivity) ^43,44^ and more accurate beat perception tasks ^45,46^ and sensorimotor synchronization abilities ^43,46–50^. Our work provides evidence for these phenotypic correlations, where people with higher genetically influenced beat synchronization may be more likely to self-select into playing a musical instrument or singing. However, we predict these genetic associations operate in parallel with the complex genetic and environmental interplay cascading throughout life. Greater genetic propensity for rhythm increases music engagement, which in turn modifies gene expression in the auditory-motor system and further heightens rhythmic abilities. Early targeted rhythm interventions might increase music engagement and further cascade the effect of active music engagement on health outcomes. Additionally, our results provide inconclusive evidence for causal associations between active music engagement and language abilities. Nevertheless, significant genetic correlations between active music engagement frequency and language skills support Nayak et al.’s (2022) hypotheses in the *Musical Abilities, Pleiotropy, Language, and Environment* framework, suggesting that the shared genetic etiology may be explained through horizontal pathways such as co-expression of genes or mediating neural mechanisms ^26^.

Our genetic correlation analyses showed that the genetic architecture of active music engagement frequency is connected to several aging-related health traits. GWAS-based genetic correlations indicated significant associations between the frequency of active music engagement and general cognitive ability. However, the magnitude was smaller than genetic correlations with language ability and beat synchronization. Our finding expands on previous observations of genetic correlations between motor, perceptual timing, and general cognitive function ^51^ and beat synchronization and general cognitive function ^17^. To complement, genetic correlations with available cognitive traits in CLSA (using bivariate-GREML) showed evidence for shared genetic etiology between active music engagement frequency and processing speed, executive function and inhibition, verbal fluency, phonological fluency, and mental flexibility. Previous phenotypic work examining has focused on the “transfer” of long-term music training to benefits in executive functioning, although many of these studies had high bias and reporting issues ^52,53^. Despite this, a meta-analysis of 9 correlational studies showed that older musicians, compared to non-musicians, have enhanced processing speed, attention, inhibition, verbal memory, verbal working memory, phonological verbal fluency, naming, flexibility, and visuospatial ability ^54^. Together, these observed “transfer” effects may be due to the many ways in which shared genetic factors give rise to musicality ^26,55,56^. Our findings provide a juxtaposition to the “transfer” hypothesis, showing that positive associations between active music engagement and cognition in aging may arise due to shared genetic covariation between cognitive traits and active music engagement. In other words, the genetic propensity for how often one *currently* engages with music may be protective of cognitive function in aging. This is impactful given that the cognitive tests in CLSA are reliable and clinically relevant for Alzheimer’s disease and dementia ^57^. Given that musicians, compared to their non-musician twins, may have lower dementia risk ^58^, future work should investigate the shared genetic and environmental interplay of lifetime active music engagement on biological and clinical dementia risk.

Bivariate-GREML analyses also revealed substantial genetic correlations between music engagement frequency and increased motor function, i.e., faster gait speed and better balance. These motor phenotypes are important for healthy aging and are predictive of frailty, falling risk, cognitive decline, and Alzheimer’s disease and dementia risk ^59–62^. Similar to cognitive literature, studies have also assessed the “transfer” of music training to enhanced motor function. For example, musicians, compared to non-musicians, have enhanced performance on standardized motor tasks, e.g., the Purdue Pegboard test ^63^ and the fingertip cross-localization test of interhemispheric function ^64^. Compared to non-musicians, musicians show faster reaction times during spatial ^65^ and multisensory integration tasks ^66^ and greater accuracy for motor sequence learning ^67,68^ and visuomotor learning ^69^. Our results complement recent work showing that genetic predispositions for better motor function, i.e., PGS for faster self-reported walking pace, was associated with greater music engagement across four cohorts (Henechowicz et al., 2025, *under review*).

Although greater genetic propensity for active music engagement frequency was associated with elevated risk for mood disorders (including depression, mania, bipolar disorder, and dysthymia), it was also associated with greater *resilience* to psychological distress (lower cumulative score on the Kessler scale). Gustavson et al. (2021) highlight this complex relationship where experimental research shows that music engagement promotes socio-emotional well-being and mental health, while on the other hand, musicians have a greater genetic and phenotypic risk for psychiatric disorders, including schizophrenia, bipolar disorder, and major depressive disorder ^27–29,70,71^. Although increased psychiatric risk may reflect the occupational stress of artistic careers ^27,29^, there is also evidence showing links between creativity and schizophrenia, including epidemiological associations ^72,73^, overlaps in neural processes ^74,75^, and shared genetic etiology ^39,76^. Alternatively, increased psychiatric risk in musicians may reflect *reverse causation,* where people who are at greater risk for mental health problems may seek out music engagement more to alleviate symptoms ^29^. Our findings could also reflect the aging-specific benefits of participating in creative, cognitively stimulating, and physical leisure activities on psychological health ^5,77^. Future work should disentangle positive and negative associations using genetic and environmental interaction models.

Lastly, prosocial behaviours (i.e., volunteering and attending religious events outside the home) showed significant genetic correlations with active music engagement frequency. Social engagement, e.g., arts, cultural activities, and volunteering, reduces isolation, loneliness, and sedentary behaviour ^10,78^, which is essential for healthy aging and Dementia prevention ^79–81^. The positive genetic correlation with religious involvement may reflect individuals who “sing in a choir,” which is a prominent part of Western European religious practises (e.g., church choirs). Additionally, the shared genetic etiology of active music engagement and increased sociality further supports evolutionary and biological research, showing that music engagement may have evolved as a form of social communication ^82,83^. The social benefits of music engagement enhance health across the lifespan as music-based interventions improve social and communication skills in children with Autism spectrum disorder and speech-language disorders ^84–88^. In older adults, community music programming and group choirs foster social connectedness, reduce isolation, and improve well-being ^89–91^.

Although our study provided novel insights into the genetic architecture of active music engagement frequency in aging, our study has limitations to consider. First, the phenotype was implemented with the intention of measuring mental exercise and contains both “playing a musical instrument” and “singing in a choir,” including two different contexts of active music engagement. Reassuringly, our analyses reveal that the phenotype measures aspects specific to musicality, given the high genetic correlation with the beat synchronization GWAS and the significant prediction of music engagement in an external cohort. However, playing an instrument and singing in a choir both have motor demands, albeit different subsystems: playing a musical instrument involves fine motor skills of the upper limbs ^92^, while singing uses a vocal motor system ^93^. Although “Play a musical instrument” does not specify the social setting, even solo music practice is social to some extent, e.g., solo music engagement is used as a tool for self-reflection and can evoke personal memories. Additionally, this measurement may exclude singers who sing on their own or in non-choir settings.

Despite limited power to detect genome-wide significant loci at the traditional threshold (*p*<5×10^−8^), our findings significantly contribute to the field with emerging evidence that active music engagement is heritable, connected to musical rhythm abilities (i.e., beat synchronization) and is a transdiagnostic indicator of healthy aging. Since lifestyle questionnaires are becoming available in large cohorts, our results provide proof of concept and pave the way for well-powered meta-GWAS studies that can further discover genetic loci. Given the top genetic locus may be implicated in affecting gene expression in the cerebellum, future work may examine associations with gene expression in specific regions of the cerebellum to understand this relationship further. It is a significant limitation that the Canadian Longitudinal Study on Aging is primarily European genetic ancestry, which limits our results in understanding the health implications for these populations^94^. It is of utmost importance to leverage multi-ancestry GWAS methods in larger cohorts with non-European ancestries and to implement music-related phenotyping into non-European ancestry cohorts to understand how music is related to health in all populations and reduce healthcare disparities ^38^. Although our two-sample MR study showed potential causal evidence for beat synchronization on more frequent active music engagement, the interpretation is limited due to the proportionally larger power of the beat synchronization GWAS and possible violation of MR assumptions. Therefore, we encourage replication with larger active music engagement GWASs and using different methodologies (e.g., twin-based or one-sample MR and structural equation modelling).

## Conclusion

In conclusion, our GWAS of a novel trait, active music engagement frequency, in CLSA revealed that the common genetic variation associated with playing a musical instrument or singing is enriched for neurobiological function. We demonstrated shared genetic etiology of active music engagement frequency to several aspects of aging-related health traits, including positive genetic correlations with cognition, motor function, language, social engagement, and mental health resilience, albeit increased risk for mood disorders. Lastly, our bi-directional MR analyses indicated that genetic propensity for musical rhythm ability may increase active music engagement frequency. Together, these findings carve the way for a new research domain of music *in* health, shifting the focus from experimental studies to studying the epidemiology of musical behaviour.

## Methods

### GWAS of Active Music Engagement Frequency

The GWAS was conducted using the generalized linear mixed model in SAIGE (version 1.1.9) ^95^ to test for associations between 8,321,422 common autosomal and X-chromosome variants (MAF>0.01 and imputation INFO>0.8) and active music engagement frequency in 23,782 individuals of European ancestry from the Canadian Longitudinal Study on Aging^96,97^ (See **Supplementary Methods** for quality control and population stratification procedures).

Covariates included were sex, age, sex×age, age^2^, and the first seven genetic principal components. SAIGE was used to account for cryptic relatedness in the sample ^95^. In fitting the null generalized linear mixed model (step 1 of SAIGE), we used a subset of 50,000 LD-pruned SNPs by performing LD pruning using PLINK2.0 ^98^ removing SNPs with r^2^ > 0.2 within 500 kb windows and 100 SNPs at a time, and selecting 50,000 SNPs at random. Association analyses (step 2) were performed on the entire sample of SNPs.

### Heritability Estimation

Heritability was calculated for active music engagement frequency based on measured SNPs (i.e., GREML) via the GCTA software tool on non-imputed genetic data (see Supplementary **Methods 2.1.1.** for quality control) ^21,99^. GCTA-GREML analyses were implemented on the maximal set of n=19,522 unrelated individuals with European ancestry, as GREML must be conducted within samples from the same ancestral background ^21,99^. Heritability estimates were calculated for the active music engagement phenotype, controlling for age, sex, and the first seven ancestry-based principal components (PCs). We used the GCTA-GREML power calculator to conduct a post-hoc power analysis ^100^.

### Post-GWAS Analyses

#### Locus Definitions and Functional Gene Mapping

The FUMA (Functional Mapping and Annotation of Genome-Wide Association Studies, https://fuma.ctglab.nl/) toolkit was used to identify lead SNPs and genomic risk loci. The lead SNP maximum *p-*value setting was set liberally to 1×10^−5^ (and all other settings were set to default) to allow SNPs to be included at suggestive significance threshold and to annotate more lead SNP ^101,102^. For SNP-to-Gene annotations, we used FUMA’s expression quantitative trait loci (eQTL) to annotate loci to genes implicated in gene expression in tissues related to neurobiological function. We set the false discovery rate threshold (FDR) at *FDR* < 0.05 to define significant eQTL associations. The eQTL databases selected were: Schwartzentruber et al.’s (2018) annotations for sensory neuron function ^103^, xQTL server of n=494 samples from dorsolateral prefrontal cortex tissues ^104^, PsychENCODE eQTLs from combined sources of the prefrontal cortex, temporal lobe, and cerebellum tissues in n=1287 individuals ^105^, eQTLs from tissues of 10 brain regions from BRAINEAC ^106^, CommonMind Consortium cis- and trans- eQTLs from post-mortem brain tissue of the dorsolateral prefrontal cortex ^107^, and GTEx v8 all 54 tissues including brain tissues of the basal ganglia (caudate nucleus, Nucleus accumbens, Substantia nigra and putamen), brain cortex, frontal cortex (BA9), anterior cingulate cortex (BA24), hippocampus, amygdala, hypothalamus, cerebellar hemisphere, cerebellum, and brain spinal cord ^36^.

#### LDSC SNP-based Heritability and Partitioned Heritability

In addition to the GCTA-GREML heritability estimates, SNP-based heritability was also calculated using LDSC and the GWAS summary statistics for active music engagement frequency in LDSC (v2.0.1) ^18^. We investigated the enrichment of the genetic architecture of active music engagement frequency in brain cell types using LDSC partitioned heritability analysis (https://github.com/bulik/ldsc/wiki/Partitioned-Heritability) with baselineLD model v2.2 and eight human genome annotations of promoter and enhancer regions of neurons, oligodendrocytes, microglia, and astrocytes ^23^. We used the Nott et al. (2019) gene set annotations because they represent cell types involved in distinct neurobiological functions. We examined the total enrichment in each category and FDR-corrected for eight tests.

#### Polygenic Score (PGS) Replication Study

We examined whether PGSs for active music engagement frequency (PGS_music_) derived from our CLSA GWAS would predict phenotypes related to active music engagement in two independent target cohorts of non-overlapping European ancestry. Specifically, we calculated PGSs in n=4,844 from the “2003–2005” wave and n=4,006 from the “2011” wave of data collection in the Wisconsin Longitudinal Study of Aging^108^ and in n= 56,216 from the Trøndelag Health Study (HUNT)^109–111^ (see**Supplementary Methods 2.2 and 2.3** for information on genetic quality control and phenotyping). PGS_music_ were calculated using the GWAS summary statistics for music engagement frequency in the target cohort using PRS-CS with reference to the European linkage disequilibrium (LD) reference panel from the 1000 Genomes Project Phase 3 European ^25,112^. PRS-CS uses a Bayesian regression framework and places a continuous shrinkage prior to SNP effect sizes ^25^, outperforming other methods, such as clumping and thresholding, in predicting complex traits ^113^. All analyses were conducted with the PRS-CS performed with the phi=0.01 as suggested for the discovery GWASs that are highly polygenic and N<100,000 ^25^. In both cohorts, PGSs were scaled to mean of 0 and standard deviation of 1 prior to analyses. In WLS, logistic regression models were conducted to assess the relationship between PGS_music_ and music engagement outcomes, covarying for age, sex, and the first ten genetic ancestry principal components. In HUNT, we conducted a linear regression model to assess the relationship between PGS_music_ and performing arts engagement, covarying for birth year, sex, and the first ten genetic ancestry principal components.

#### Genetic Correlation Analyses

Genetic correlation analyses were designed to investigate the shared genetic variation between active music engagement frequency and health traits. We examined genetic correlations using two different methods. First, we investigated genetic correlations using bivariate LDSC (v2.0.1) ^18^ with the GWAS summary statistics for active music engagement frequency and 24 summary statistics including psychiatric, neurodegenerative and aging, motor, and cognitive and language traits and the GWAS of beat synchronization in n=606,825, which is the largest existing GWAS of a musicality trait. See Supplementary **Table 1** for details on the GWASs included for LDSC-genetic correlations.

Given that the GWASs of different health traits were conducted in cohorts with different demographics, we also applied bivariate-GREML, which uses individual-level data (rather than summary-level data) to investigate genetic correlations ^19^ between active music engagement frequency and 22 traits within the CLSA cohort measuring cognition, motor function, physical activity, psychiatric and mental health, substance use, and social participation (See **Supplementary Table 2** for details on the phenotypes included in bivariate-GREML genetic correlations). This allowed us to see the shared genetic variation between active music engagement and health traits in the same age resolution. We applied FDR correction to both genetic correlation analyses separately to account for multiple tests.

#### Two-Sample Mendelian Randomization

As a follow-up, we conducted bidirectional two-sample MR studies with music engagement frequency and traits significantly genetically correlated with active music engagement frequency. All analyses were performed using the *TwoSampleMR* R package ^114^. Instrumental variables (SNPs) were selected using the F-statistic>10 and *p*<5×10^−6^. We selected non-correlated SNPs by clumping SNPs with R2<0.01 and in 1000kb windows with reference to 1000 Genomes European population ^112^. Since the threshold of *p*<×10^−6^ only yielded 8 SNPs for active music engagement frequency as the exposure, we relaxed the instrument threshold to *p*<5×10^−5^ for the active music engagement frequency GWAS to ensure sufficient numbers of SNPs ^41^. We conducted two-sample MR analyses using the inverse variance weighting method and MR-Egger regression. We conducted a heterozygosity test, pleiotropy test, Steiger test, leave-one-out analyses, and outlier tests for sensitivity analyses. We applied Bonferroni correction for multiple testing corrections for eight tests.

## Supplementary Material

Supplementary Files

This is a list of supplementary files associated with this preprint. Click to download.

• HenechowiczTaraL202506AppendixAtables.xlsx

• SupplementaryforDUAOctober102025.docx

## Figures and Tables

**Figure 1:**
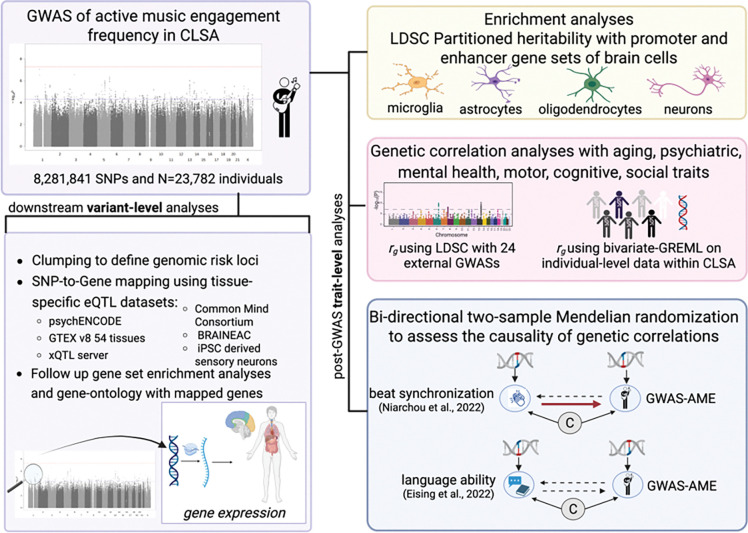
Study Overview

**Table 1.** Suggestive Genetic Loci Associated with Active Music Engagement Frequency

Genomic locus	Rsid	CHR	POS	BETA	SE	EA	NEA	EAF	*p*-value	eQTL mapped gene(s) (tissue)
1	rs7554669	1	85880933	−0.07	0.01	A	G	0.18	9.2×10^−8^	*DDAH1* (GTEx Liver v8); *RP11-131L23.1* (GTEx v8 Brain Cerebellum*; GTEx v8 Skin sun exposed lower leg)
6	rs9647401	3	73182547	−0.06	0.01	G	A	0.23	2.8×10^−7^	N/A
9	rs3805476	5	172195092	0.07	0.01	A	G	0.15	4.3×10^−7^	*RP11-779018.1* (GTEx/v8/Skin_Sun_Exposed_Lower_leg); *RP11-779018.2* (GTEx/v8:Artery Tibial); *RP11-779018.3*(GTEx/v8:Whole Blood; GTEx/v8 Spleen; GTEx v8 Testis)
14	rs80055245	13	66534731	0.15	0.03	C	T	0.03	9.3×10^−7^	N/A
8	rs10044788	5	103551497	0.12	0.02	C	T	0.05	9.6×10^−7^	N/A
15	rs79292477	14	26668335	−0.11	0.02	A	T	0.05	1.5×10^−6^	N/A
2	rs12729624	1	168930460	0.06	0.01	T	C	0.21	2.2×10^−6^	*ATP1B1* (GTEx v8 Minor Salivary Gland); *AL021068.1* (CMC_SVA_cis)* *RPL29P7* (GTEx v8 Testis)
3	rs114029967	1	235439317	−0.19	0.04	C	T	0.02	2.5×10^−6^	*TBCE* (GTEx v8 Heart Atrial Appendage); GGPS7(GTEx v8 Lung; GTEx v8 Tibial Nerve); *B3GALNT2* (GTEx v8 Thyroid)
7	rs16885512	5	55766621	0.09	0.02	G	C	0.08	2.5×10^−6^	*CTC-236F12.4* (GTEx v8 Thyroid)
20	rs5986261	23	25712567	0.17	0.04	T	G	0.01	3.1×10^−6^	N/A
19	rs6124907	20	45607859	−0.05	0.01	A	C	0.34	3.3×10^−6^	N/A
18	rs117450000	18	64674501	0.14	0.03	G	A	0.03	4.2×10^−6^	N/A
4	rs116481454	3	3687572	0.10	0.02	C	G	0.05	5.7×10^−6^	*LRRN1* (GTEx v8 Sun not exposed suprapubic)
21	rs5924107	23	87002770	0.07	0.02	G	A	0.08	5.8×10^−6^	N/A
5	rs115067899	3	16537969	−0.15	0.03	T	C	0.02	6.0×10^−6^	N/A
13	rs143824048	9	114591558	0.18	0.04	A	G	0.02	6.1×10^−6^	*GNG10*(PsychENCODE* GTEx Artery v8 Tibial; GTEx v8 Esophagus Muscularis; GTEx v8 Muscle Skeletal; GTEx v8 Nerve Tibial; GTEx v8 Thyroid)
Genomic locus	Rsid	CHR	POS	BETA	SE	EA	NEA	EAF	*p*-value	eQTL mapped gene(s) (tissue)
17	rs4572341	15	86003010	0.08	0.02	A	G	0.09	7.3×10^−6^	*RP11-561C5.4* (PsychENCODE*; GTEx v8 Adipose Visceral Omentum; GTEx v8 Lung; GTEx v8 Muscle Skeletal); *RP11-815J21.3* (GTEx v8 Testis); *CSPG4P12* (GTEx/v8/Muscle_Skeletal); *RP11-158M2.5* (GTEx v8 Skin sun exposed lower leg; GTEx v8 Testis); *CTD-2262B20.1* (GTEx v8 Skin not sun exposed suprapubic); *RP11-158M2.4* (GTEx v8 Espohagus Mucosa; GTEx v8 Skin sun exposed lower leg)
10	rs6904638	6	541668	0.06	0.01	T	C	0.18	7.4×10^−6^	*EXOC2* (PsychENCODE; CMC_SVA_cis* GTEx v8 Esophagus Mucosa; GTEx v8 Muscle Skeletal; GTEx v8 Cells Cultured fibroblasts); *RP11-532F6.3* (PsychENCODE)*
16	rs10145529	14	33367794	−0.05	0.01	T	A	0.39	8.5×10^−6^	N/A
12	rs113114385	7	31685507	0.16	0.04	C	A	0.02	9.2×10^−6^	N/A
11	rs2765233	6	67242496	−0.05	0.01	T	C	0.25	1.0×10^−5^	N/A

*Note*. The suggestive genetic loci were identified using FUMA SNP-to-Gene mapping and are ordered by the GWAS *p*-value of the lead SNP. ‘Rsid’: rsid for thelead SNP, ‘CHR’: chromosome, ‘POS’: position in GRCh37/hg19, ‘BETA’: the effect, ‘EA’: effect allele, ‘NEA’: non-effect allele, ‘EAF’: effect allele frequency, ‘SE’: standard error, ‘p-value’: GWAS *p*-value, ‘eQTL mapped gene(s)’: genes mapped using eQTL databases with database and tissue in brackets (see Locus definitions and functional gene mapping for methods).

(*) The **bolded text** indicates genes mapped from eQTLs that modulate gene expression in brain tissues.

**Table 2.** Bidirectional Two-sample Mendelian Randomization Results

Exposure	Outcome	Method (*p*-value thresh.)	Primary analyses			Sensitivity tests			Outlier analyses		
			N_SNP_	*b* [95%CI]	*p*	Het	Pleio (*p*)	Steiger test (*p*)	N_SNP_	*b* [95%CI]	*p*
ME	beat-sync	MR-Egger (5e-6)	10	0.03 [−0.26,0.32]	0.83	Q=12, *p*=0.17	0.62	4.3e-47	9	0.17 [−0.17,0.50]	0.36
ME	beat-sync	INV-W (5e-6)	10	0.10 [0.01,0.20]	0.03	Q=12, *p*=0.21		4.3e-47	9	0.13 [0.03,0.22]	0.007
ME	beat-sync	MR-Egger (5e-5)	106	0.05 [−0.04,0.14]	0.26	Q=155, *p*=0.001	0.79	0	100	0.08 [−0.01,0.17]	0.07
ME	beat-sync	INV-W (5e-5)	106	0.04 [0.00,0.08]	0.03	Q=155, *p*=0.001		0	100	0.05 [0.01,0.08]	0.006
beat-sync	ME	MR-Egger	61	0.31 [−0.14,0.77]	0.18	Q=66, *p*=0.25	0.44	0.87	59	0.18 [−0.27,0.63]	0.44
**beat-sync**	**ME**	**INV-W**	**61**	**0.14 [0.07,0.20]**	**4.2e-5** *	**Q=67, *p*=0.26**		**0.87**	**59**	**0.13 [0.07,0.19]**	**6.1e-5**
ME	language	MR-Egger (5e-6)	9	−0.14 [−0.41,0.13]	0.34	Q=7, *p*=0.43	0.19	2.4e-17	8	−0.10 [−0.41,0.20]	0.53
ME	language	INV-W (5e-6)	9	0.05 [−0.06,0.15]	0.39	Q=9.2, *p*=0.33		2.4e-17	8	0.06 [−0.05,0.17]	0.26
ME	language	MR-Egger (5e-5)	86	0 [−0.09,0.10]	1.0	Q=101, *p*=0.09	0.32	1.9e-109	79	−0.02 [−0.11,0.07]	0.70
ME	language	INV-W (5e-5)	86	0.04 [0.01,0.08]	0.03	Q=103, *p*=0.09		1.9e-109	79	0.04 [0.00,0.08]	0.03
language	ME	MR-Egger (5e-6)	28	0.31 [−0.11,0.72]	0.16	Q=35, *p*=0.12	0.21	1.5e-40	25	0.19 [−0.30,0.68]	0.45
language.	ME	INV-W (5e-6)	28	0.05 [−0.07,0.17]	0.43	Q=37, *p*=0.10		1.5e-40	25	0.02 [−0.10,0.14]	0.78

*Note.* ME=active music engagement frequency, beat-synch=beat synchronization.

(*) denotes significance after Bonferroni correction.

Het=Heterozygosity test result, Pleio=Test for horizontal pleiotropy. INV-W=Inverse variance weighted meta-regression. Method (*p*-value thresh.) = method used for MR meta-analyses and the *p*-value threshold for selecting instruments; if the *p*-value threshold is not specified, the *p*<5×10^−8^ threshold was used.

## Data Availability

Individual data are available from the Canadian Longitudinal Study on Aging (www.clsa-elcv.ca) for researchers who meet the criteria for access to de-identified CLSA data. Individual data from the Wisconsin Longitudinal Study are available for researchers who meet the criteria to access de-identified data. Data from the HUNT Study may be accessed by application to the HUNT Research Centre. PMIDs for GWAS summary statistics are available in **Supplementary Table 1.** The full GWAS summary statistics from the original study of musical beat synchronization (23andMe discovery studies set) have been made available through 23andMe to qualified researchers under an agreement with 23andMe that protects the privacy of the 23andMe participants. Datasets will be made available at no cost for academic use. Please visit https://research.23andme.com/collaborate/#dataset-access/ for more information and to apply to access the data. Participants provided informed consent and volunteered to participate in the research online, under a protocol approved by the external AAHRPP-accredited institutional review board, Ethical and Independent Review Services. As of 2022, Ethical and Independent Review Services is part of Salus institutional review board (https://www.versiticlinicaltrials.org/salusirb). All code for this project will be deposited on Open Science Framework.
